# Analyzing appropriate autonomous vessel for South-East Asian route: from the view of seafarers

**DOI:** 10.1186/s41072-022-00122-9

**Published:** 2022-09-01

**Authors:** Bornali Rahman

**Affiliations:** Shipping and Maritime Science, Canadian University of Bangladesh, Dhaka, Bangladesh

**Keywords:** Autonomous vessel, South-East Asia, Accident, AHP, MCDA

## Abstract

The development of autonomous vessel has achieved tremendous interest across the world for the safe navigation and economic benefits. Numerous alternatives are constructed in the autonomous vessel development projects, the alternatives of MUNIN and NYK project are combined for this study; these are - Manned autonomous vessel, Remotely controlled vessel, Autonomous and Partially remote-controlled vessel, and Full autonomous vessel. As the statistics of UNCTAD shows that South-East Asia is a highly dense region and has the busiest international maritime connectivity, this research tried to select the appropriate autonomous vessels from the four alternatives to ensure safe navigation in this traffic congested maritime route. For this study, 311 accident reports are investigated to find out the most frequent casualty and its cause. The data are collected from the global integrated shipping information system of the international maritime organization's website. The decision tree of R-studio demonstrates that the most frequent accidents are- Collision, Grounding, Fire, and listing. Afterwards a survey was made on 65 experienced seafarers to determine which autonomous vessel criteria would be compatible to avoid the casualty. This research adopts AHP (analytical hierarchy process) to conduct the analysis. AHP is a multi-criteria decision analysis method for solving any decision problem. The research shows that ‘Manned autonomous vessel’ and ‘Autonomous and Partially remote-controlled vessel’ are the appropriate alternatives for safe navigation in the South-East Asian region. This study will help the researcher who is working in autonomous vessel development, mainly working for Asian water.

## Introduction

The increasing trends of technology have been reforming our present world; now we are sailing towards the fourth industrial revolution where AI application has taken the most crucial role in the industrial revolution. Following the emerging digitalization, the shipping industry has started stepping towards autonomous shipping. Globally ocean is considered a role player in goods transportation, energy exploration, and tourism. World’s more than 80% of cargoes are shipped by sea (UNCTAD 2018). It has been seen that; human error is responsible for around 70–90% marine accident (Flokkou 2021). Such accident results life loss, cargo loss, financial loss and environment pollution. With a view to enhancing the vessel competitiveness and eliminating human error onboard, the concept of the autonomous vessel has been established which has enormous benefits. The reduction in the vessel’s operation costs and integrated navigational safety are the main benefits of autonomous vessel (Kurt and Aymelek 2022). Due to this reason, shipping companies are interested in the launching of autonomous vessel. It’s estimated that, the autonomous ship market is 5866 million USD and it’s predicted that, the share will be 14,256 million by 2030 (Research and Markets. Global Autonomous Ships Market (2020 to 2030) - Increasing [Internet]. 2021 [cited 2021 Dec 31]. Available from:https://www.globenewswire.com/en/news-release/2021/01/21/2162032/28124/en/Global-Autonomous-Ships-Market-2020-to-2030-Increasing-Use-of-Automated-Systems-to-Reduce-Human-Errors-and-Risks-is-Driving-Growth.html^2020^). In Europe, numerous projects are being developed for autonomous implementation, such as Kongsberg Maritime, Maritime Unmanned Navigation through Intelligence in Networks MUNIN project by European Commission, The Autonomous Waterborne Applications Initiative (AAWA) project by Rolls-Royce, Re-volt by DNV GL, Cyber‐enabled ship project by Lloyd’s Register (LR), Autonomous Marine Operations and Systems (AMOS) by the Norwegian University of Science and Technology (NTNU) (Komianos 2018). Wartsila, NYK project, Unmanned Cargo Ship Development Alliance are the Asian projects for autonomous ships. (Wee 2021; Liang 2019; SAFETY4SEA. China launches unmanned cargo ship development alliance [Internet]. 2017). Norway acts as the pioneer for the implementation of the autonomous vessels, along with this Finland, Uk, USA, China, Japan, Singapore, Hongkong, and a few other countries are also working on this project (SAFETY4SEA. IR Class launches guidelines for autonomous and remotely operated vessels [Internet]. 2021). The first autonomous vessel grounded on the water in 2018 by the collaboration of Wilhelmsen and Kongsberg, the autonomous boat SEA-KIT,12 m long, transshipped cargo of oysters from West Mersea of the UK to Oostende, Belgium in 2019 which is considered as the first autonomous commercial vessel of the North Sea (SAFETY4SEA. Autonomous ships: Test areas and research centers making headlines [Internet]. 2019).The project development shows that, the launching of autonomous vessel is not so far, and this will connect several maritime routes across the world (Kurt and Aymelek 2022). Asia plays a vital role in worlds economy, the UNCTAD report shows that, in 2020 around 40% export and 65% import of world’s total trade takes place in this region (UNCTAD 2021). The South-East Asia has been considered as the main export region in the world, which consists the mainland Southeast Asia and a variety of archipelagoes to the south and east of this location. The region is connected with the long sea routes like European and U.S.A market (Simões Toccheto et al. 2014). As per the statistics of UNCTAD, South-East Asia is a highly dense region and has the busiest international maritime connectivity (UNCTAD 2021). In order to ensure efficient and safe navigation without any human error in this traffic congested region, autonomous vessel can be considered as a suitable navigation mode, due to the low operation cost and safe navigation properties.

The autonomous vessel can be classified in several types. The classification is varied according to projects. In this research, the author tried to analyze the appropriate vessel for the South-East Asian route focusing on the most frequent accident criteria. For this study, 311 accident cases of Indonesia, Malaysia, Singapore, Vietnam, Hongkong, and China (adjacent to the South China Sea) coasts are analyzed to demonstrate the frequency of different types of casualties in south-east Asia. Furthermore, questionnaire surveys were conducted among the seafarers to find an appropriate autonomous vessel alternative that can ensure safe navigation in this region. The accident data was collected from GISIS of IMO and research is conducted by quantitative analysis using AHP -MCDA model through R studio and Python3 program. This research can contribute to the autonomous vessel's operational development, especially in the implementation of the autonomous vessel in Asian water. In addition, the study can also suggest several factors that can be taken into account during the construction of the autonomous vessel regulation.

The “Introduction” has familiarized with the autonomous vessel and briefed the importance of South-East Asia, the “Literature review” described several literatures related to the autonomous vessel and explained the significancy of the current study. "Methodology" clarified the research procedure, "Analysis of Autonomous vessel for South-East Asia's Maritime Route" assessed the alternatives and find out the appropriate autonomous vessel for safe navigation in South-East Asian region. The "Discussion" section discussed the result of analysis. Finally, the "Recommendation and Conclusion" section concludes the research suggesting several factors which are required to implement the autonomous vessel in the South-East Asian region and concludes the research.

## Literature review

Autonomous vessel can implement an innovative business model cutting the cost and enhancing the efficiency, due to this reason, this concept has achieved great concentration in commercial shipping (Munim 2019). The concept has gathered maximum attention among the researchers also, as this emerging concept will take the maritime industry towards the fourth industrial revolution. Many aspects of autonomous vessel have been studied by the researchers worldwide. Wróbel et al. (2020) studied on the research direction of remote-controlled autonomous vessel using the ‘System Theoretic Process Analysis (STPA)’.In this research the author reviled that most of the autonomous researches are related to the technical function where organizational and human issues are overlooked (Wróbel et al. 2020). Karlis Thanasis (2018) in his research ‘Maritime law issues related to the operation of unmanned autonomous cargo ships ‘describes about the important sector of existing maritime law and regulation, which should be revised in accordance with the autonomous vessel’s operational function (Karlis 2018). The study focused on the organizational function related to the autonomous vessel, the competitiveness of the vessel is not explained in this study. Another researcher Christos Flokkou (2021) described about the alternatives of the autonomous vessel, along this explained the possible challenges (Flokkou 2021). The study is qualitative research where the solutions to mitigate the challenges are also suggested. Though the research broadly described about different levels of autonomous vessel, the appropriate one is not identified (Flokkou 2021). Munim, Ziaul Haque in his paper ‘Autonomous ships for container shipping in the Arctic routes’ analyzed the competitiveness of several autonomous vessels and find out the appropriate vessel for the arctic region using the opinions of the expertise, in this research he used MCDM technique for data analysis (Munim et al. 2020). The study covers important criteria for shipping, but didn’t emphasize the collision avoidance ability, which is crucial for safe navigation. This research gap encouraged the author to conduct a study on the appropriate autonomous vessel for one of the world’s busiest maritime trade regions South-East Asia, based on the collision avoidance capability. As ship and seafarers both are perceived as the prior element for ship management (Utureanu and Cristina 2016), the author collected the seafarers’ opinion in this study. This study aims to find out the best suitable autonomous vessel among four alternatives for safe navigation in Southeast Asia. The research comprised the three important elements – Autonomous vessel, South-East Asia’s maritime route and opinion of seafarers. Reviewing the existing literatures, author has found that none of the research has worked on the arena of this current study.

### Autonomous vessel

The autonomous vessel has achieved tremendous interest worldwide; in line with this, many researchers have shown their interest in autonomous vessel development. The vital factor of this research is to analyze surroundings and monitor own health condition (Vtt 2016). Automation is such a process equipped with a machine and AI control that can replace humans in any operation (Bureau of Shipping 2021). The definition of the autonomous vessel is differ according to project. The Norwegian Forum for Autonomous Ships (NFAS) defined autonomous ship as a computerized vessel which is able to operate without any human interference (Rodseth and Nordahl 2017). Maritime Unmanned Navigation through Intelligence in Networks (MUNIN) project of the European Commission, explained that, the autonomous ship is the application of advanced control and communication technology in the vessel which will provide the capability of operating the vessel remotely, semi autonomously or full autonomously (MUNIN 2016). The Maritime Safety Committee (MSC) of the International Maritime Organization (IMO) stated the autonomous vessel as ‘Maritime Autonomous Surface Ship (MASS)’, which can be operated without any human interaction in a varying degree (IMO. MSC 99/22 [Internet]. 2018). The definition has not explained the operational procedure of ‘Maritime Autonomous Surface Ship’. Analyzing several projects and properties of the autonomous vessel, we can define that, the autonomous vessel is such vessel in which the application of Artificial Intelligence (AI) and IoT (Internet of Things) will enable the vessels to operate without any human interference in varying modes.

#### Operational properties of autonomous vessels

Human participation is not required in the autonomous vessel, for this reason, the total decision making is carried out by using the algorithm. Analyzing the situation, a vessel can avoid collision using the planning based on the developed set of electronic senses for machine learning. The algorithm plays a vital role in autonomous operation, acting as the human brain and eliminating human error during navigation. Throughout the voyage, the vessel must cope with several uncertain situations and casualties where seafarers apply their own knowledge with experiences to prevent the accident. The NYK project demonstrated the technical procedure in four steps- (1) Information Acquisition. (2) Analysis. (3) Planning. (4) Approval (Koji Kutsuna et al. 2018). The entire process can be conducted by AI or remote, as well as a mixture of both. Several sensor systems are required to distinguish the complex environment, such as unknown or unanticipated objects, lousy weather, and casualty risk. The project was designed in such a way to prevent the collusion, an autonomous control will be applied to prevent the collision. When the autonomous system fails to prevent the event, the system will request the remote operator and move to the 'fail to safe' step during the absence of the remote controller. The effective collision prevention procedure of the autonomy process can narrow down the importance of human attendance in safe navigation (PARTNER| MUNIN [INTERNET]. [CITED 2021 DEC 28]. available from: http://www.unmanned-ship.org/munin/partner/2021).

The sensor system to detect the casualty is the main challenge of the autonomous concept. Electronic senses are required to develop for the electronic brain to ensure navigational safety and collision prevention. As per the AAWA (Advanced Autonomous Waterborne Applications Initiative), led by Rolls Royce, three significant areas for the Autonomous vessel are (Vtt 2016)-

*Sensor fusion*: The Sensor technology, which has remarkable application in the autonomous car, is developing for vessels also. AAWA project has explored different sensors to accumulate several pieces of information, such as radars, high-definition visual cameras, thermal imaging, and LIDAR (Light Detection and Ranging), which are essential to analyze the vessel’s surroundings in any event.

*Control algorithms*: In order to maintain safe navigation and prevent a collision at sea, the vessel needs to take appropriate action if the case exists or is in doubt. The decision algorithms which are applied for the machine learning need to be perfect and follow the 'International Convention for prevention of collision at sea”. Due to this reason, algorithm development is the crucial and challenging part of the vessel.

*Communication and connectivity*: Adequate connection capacity for vessel monitoring and remote control is essential in the autonomous vessel. The ship sensors need to be enabled to establish proper connection with satellite and land-based systems.

#### Different types of autonomous vessel

The MASS can be operating at several degrees; during a single voyage, the vessel can operate under one or more degrees (IMO takes first steps to address autonomous ships [Internet]. 2021). The different project defines different degrees or types of the autonomous vessel. The Table 1 presents different autonomous vessel alternatives, reviewing different literature pieces.

**Table 1:** The degrees of autonomy

IMO	Rolls royce	MUNIN	NYK
Ship with automated processes and decision support	Manned autonomous and remote control	Manned ship	Manned autonomous
Remotely controlled ship with seafarers on board	Remote-controlled unmanned coastal vessel	Remote ship	Remote
Remotely controlled ship with seafarers on board	Remote-controlled unmanned ocean-going ship	Automated ship	Unmanned autonomous
Remotely controlled ship with seafarers on board	Autonomous unmanned ocean-going ship	Autonomous ship	

IMO has not emphasized the AI application solely, rather than the alternatives are on-based remote sensor programs more. However, the regulation for safe autonomous vessel navigation does not provide clear guidelines (Partner | MUNIN [Internet]. [cited 2021 Dec 28]. Available from: http://www.unmanned-ship.org/munin/partner/2021). NYK and Rolls Royce developed similar autonomous criteria: Manned Autonomous, Remote Autonomous, and Unmanned Autonomous. For this study, we have combined the autonomous alternative of the MUNIN project and the Combined project to choose the appropriate autonomous modes for South-East Asian Routes. The following autonomous options are applied in MCDA-AHP method.

Manned autonomous: Where seafarers will be on board with autonomous equipment, and action will be taken manually. AI application will suggest decision making; onboard seafarers will execute and monitor the circumstances. No remote operation is required under such autonomous system.
Remotely controlled vessel: Under this alternative, all navigation operations will be performed via a remote-control mechanism
Autonomous and partially remote-controlled vessel: The vessel will be operated through remote control where AI applied equipment will be onboard.
4.Full autonomous vessel: The vessel will be artificially intelligent, and appraisal, planning, execution, and monitoring, the fundamental steps, will be conducted under the supervision of AI.

### Autonomous vessel for South-East Asia’s route

The south-east Asian region is considered as the center of gravity for Indro-pacific connectivity as the geographical properties construct the connectivity network for the Indian and Pacific oceans routes (Wróbel et al. 2020).This region historically engaged in two types of maritime trade exchange: intra-Asian and intra-regional (Shimada 2019). In order to trade the products from the production area to nearby markets, local people created the network with transit ports and thus encouraged the foreign traders to enter this region in the beginning (Ota 2019). In the current stage, this region is also the center for exporting manufacture product trading, and geographical position enabled this area to be the connector of the foreign market (Ota 2019). In such a significant route, autonomous vessel implementation ensuring safe navigation can be challenging. The geographical location of the Southeast Asia region is strategically situated at the passage of the Indian Ocean and the South China Sea (Idris and Ramli 2018), where oceans and straits constructed one of the most influential global maritime routess (IMO takes first steps to address autonomous ships [Internet]. 2021).In the early fifth century, foreign traders established the international maritime route through the Malacca straight (Hall 2019). Generally, in the past, two factors acted behind the external trade exchange: a riverine political system and the supply of surplus products from Southeast Asian mainland and Java for the foreign trader (Kenneth 2019). The route was the part of the ancient silk road and now included in the twenty-first century's silk road which begins from the Quanzhou in Fujian province, runs through Guangzhou, Beihai, and Haikou, following those heads to the south of the Malacca Straits and from the Kuala Lumpur goes towards Kolkata, India and after passing the Indian Ocean will move to Nairobi, Kenya, and the route will end at the land-based silk road in Venice after crossing the Red Sea and the Mediterranean Sea (Hong 2015). With the development of the Maritime Silk Road initiative, where Southeast Asia is one of the dominant regions, maritime traffic along the route increased significantly (Mou et al. 2021).On the other hand, Asian developed countries are accountable for the majority of world maritime trade; it is estimated that 76% of total maritime trade volume is loaded and unloaded in developing countries, and continuously the volume is increasing due to the growing container trade for the world's factory boosting in intra-Asia, especially in South-east Asia (UNCTAD 2020). As per UNCTAD, 16 of the world's top 20 container ports are located in Asia, and the positions are the same in 2018 and 2019 (UNCTAD 2020). Among them are Singapore ports, eight ports are from mainland China, one from Hongkong, and one is China. The report indicates that the south-east Asian ports welcome a large number of vessels for large volume shipment, thus resultant in dense traffic in the South-East Asian region. The maritime route of South-East Asia's route included Sumatra strait, Malacca Strait, Singapore Strait, Taiwan Strait. Geographically the entire region is congested for navigation and witnesses several maritime casualties, due to this reason, navigation with special caution is necessary. However, the autonomous vessel has such potential which can reduce human error related accidents (Porathe et al. 2018). Human contribution and control system will be reformed in this vessel technology (Mallam et al. 2020). Comparing with the traditional vessel, the operation of autonomous vessel will have less human interference (Abilio Ramos et al. 2019).The application of Artificial Intelligence (AI) and Internet of Things (IoT) can enhance the operational structure of vessel navigation and establish safety of navigation by eliminating human error and advance decision-making ability, which is important for moving in the South-East Asian region. On the other hand, the autonomous vessel is capable of bringing economic advantage in maritime business (Ziajka-Poznańska and Montewka 2021). Kreteschman L (2017) in his research conducted economic exploratory analysis on the autonomous and conventional bulk carrier, where he showed that the autonomous ship may has optimistic effect on the cost-effectiveness of shipping companies (Kretschmann et al. 2017). Akbar A (2021) stated that the autonomous vessel in short sea shipping can reduce the operating cost on average 11% (Akbar et al. 2021). The prediction of economic and safety benefits of the autonomous vessel put her at the center of concentration among the maritime business. The launching of the autonomous vessel in the South-East Asian region can provide safe and economic navigation in this important route.

## Methodology

In order to conduct research, a quantitative research procedure is adapted where a structured scale questionnaire was applied in the data. Secondary data was collected from GISIS of IMO. Snowball sampling was applied to draw the sample, for this research, experienced seafarers having proper navigation knowledge were nominated as a population. Multi Criteria Decision Analysis (MCDA) technique has been used in this research to identify the best fit autonomous vessel in the South-East Asian region.

*Multi-Criteria Decision Analysis (MCDA)* MCDA is structured and transparent decision analysis tool which is used to assist the group of individual decisionmaker taking their decision in a complex situation (Martyn JDr).The Analytical Hierarchical Process (AHP) is one famous MCDA method, which is used for pair comparison to model a problem through hierarchic or a network structure (Saaty 1987). The model is established by saaty in 1980 to calculate the weight of the criteria (Liang et al. 2017). In discrete case, the comparison will be dominance matrices and from this the ratio scales are derived into principal eigenvectors and eigenfunctions, the matrices will be positive and reciprocal (Saaty 1987).

Suppose, n types of criteria are in on hierarchy, following the pair wise comparison method of Saaty, the A matrix is derived-

1 $${\text{A}} = \left|\begin{array}{*{20}l}1 \hfill & {a_{12} } \hfill &\cdots \hfill & {a_{1n} } \hfill \\ {a_{21} } \hfill & 1 \hfill & \cdots \hfill & {a_{2n} } \hfill \\ \vdots \hfill & \cdots \hfill & \ddots \hfill & \vdots \hfill \\ {a_{n1} } \hfill & {a_{n2} } \hfill & \cdots \hfill & 1 \hfill \\ \end{array}\right |$$

Let *a*_*ij*_ represents the relative importance of criteria *i* comparing with *j*, and it can be calculated by Eq. 2,

2 $$a_{ji} = \frac{1}{{a_{ij} }},a_{ij} > \, 0, \;i, \, j = \, 1,2,...,n$$

The Eq. 3 is used to establish the hierarchy.

3 $$A =\left| \begin{array}{*{20}l} 1 \hfill & {a_{12} } \hfill & \cdots \hfill & {a_{1n} } \hfill \\ {a_{21} } \hfill & 1 \hfill & \cdots \hfill & {a_{2n} } \hfill \\ \vdots \hfill & \cdots \hfill & \ddots \hfill & \vdots \hfill \\ {a_{n1} } \hfill & {a_{n2} } \hfill & \cdots \hfill & 1 \hfill \\ \end{array} \right|\;\;\;\;\left|\begin{array}{*{20}l} {w_{1} } \hfill \\ {w_{2} } \hfill \\ \vdots \hfill \\ {w_{n} } \hfill \\ \end{array}\right| = \lambda_{\max } \left|\begin{array}{*{20}l} {w_{1} } \hfill \\ {w_{2} } \hfill \\ \vdots \hfill \\ {w_{n} } \hfill \\ \end{array} \right|$$

In the Eq. 3, (*w*_*1,*_* w*_*2*_*,…, w*_*n*)_ is the maximal eigenvector of matrix A and ${\uplambda }_{\mathrm{max}}$ is the maximal eigenvalue of matrix A.

The weighting coefficient can be calculated from the Eq. 4 by normalizing the maximal eigenvector

4 $${\text{W}} = \left( {\frac{{W_{1} }}{{\mathop \sum \nolimits_{i = 1}^{n} w_{i} }} , \frac{{w_{2} }}{{\mathop \sum \nolimits_{i = 1}^{n} w_{i} }} , \ldots , \frac{{w_{n} }}{{\mathop \sum \nolimits_{i = 1}^{n} w_{i} }}} \right)$$

In order to judge the consistency of the matrix, the Eq. 5 will be applied, if the CR < 0.1, the matrix is consistent.

5 $$CR = \frac{CI}{{RI}}$$

6 $$CI = \frac{{\lambda_{\max } - n}}{n - 1}$$

CI will be derived from Eq. 6 and the RI can be obtained from the Table 2

**Table 2:** CI table source (Saaty 1987)

Matrix size	Random consistency index (CI)
1	0.00
2	0.00
3	0.58
4	0.90
5	1.12
6	1.24
7	1.32
8	1.41
9	1.45
10	1.49

The comprehensive research is conducted under several steps using Rstudio and Python programming. A decision tree was derived from R studio to identify the most frequent casualty of 311 casualty cases of South-Eastern Asia's maritime region. Afterward, descriptive statistics assessed the causes of casualty. The casualty frequency is applied in the Analytical Hierarchy Process (AHP) to calculate the weight and rank the most suitable autonomous vessel as per the opinion of the seafarers.

## Assessment of the casualty cases in South-East Asia

In this study, 311 cases are assessed between the years 2000 and 2020 near Indonesia, Malaysia, Singapore, Thailand, Vietnam, Taiwan, Hongkong, and the southeast region of China.

**Fig. 1:**
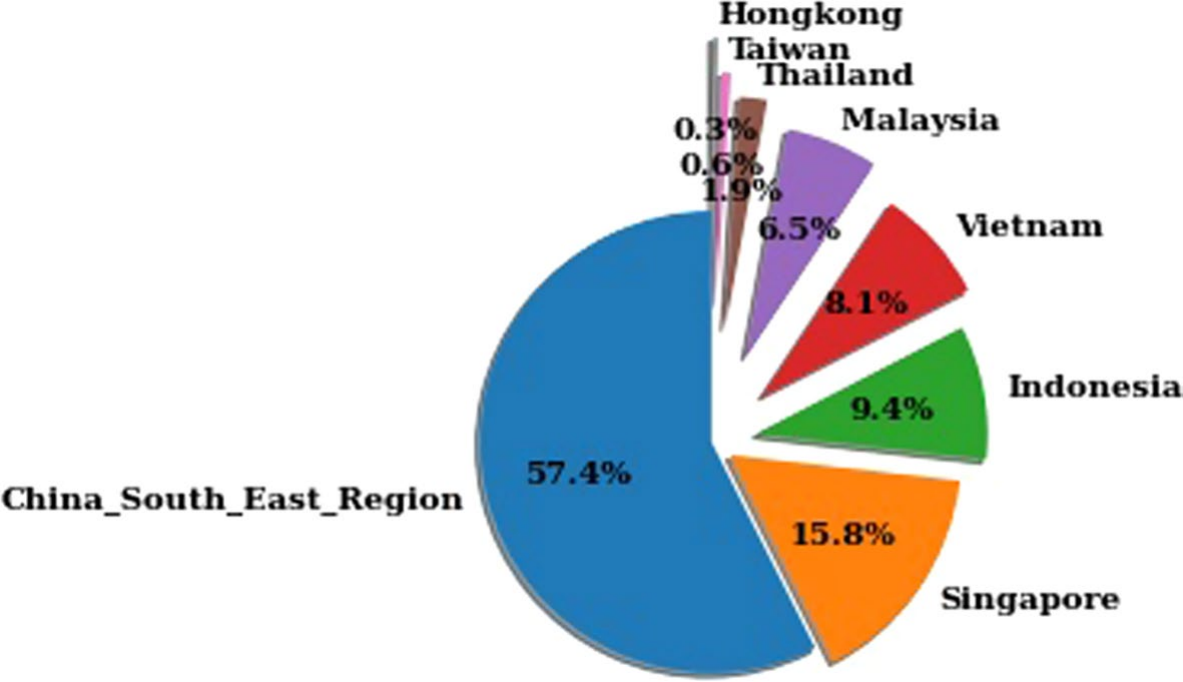
Maritime casualty in South-East Asia's coast

Figure 1 represents the percentage of maritime casualty as per country and region. The pie chart shows that 57.4% of cases are from China, and the second-highest rate is 15.8% from Singapore, where Indonesia and Vietnam are at the nearest similar rate. Though the overall shows that, Hongkong has the lowest number of accidents.

**Fig. 2:**
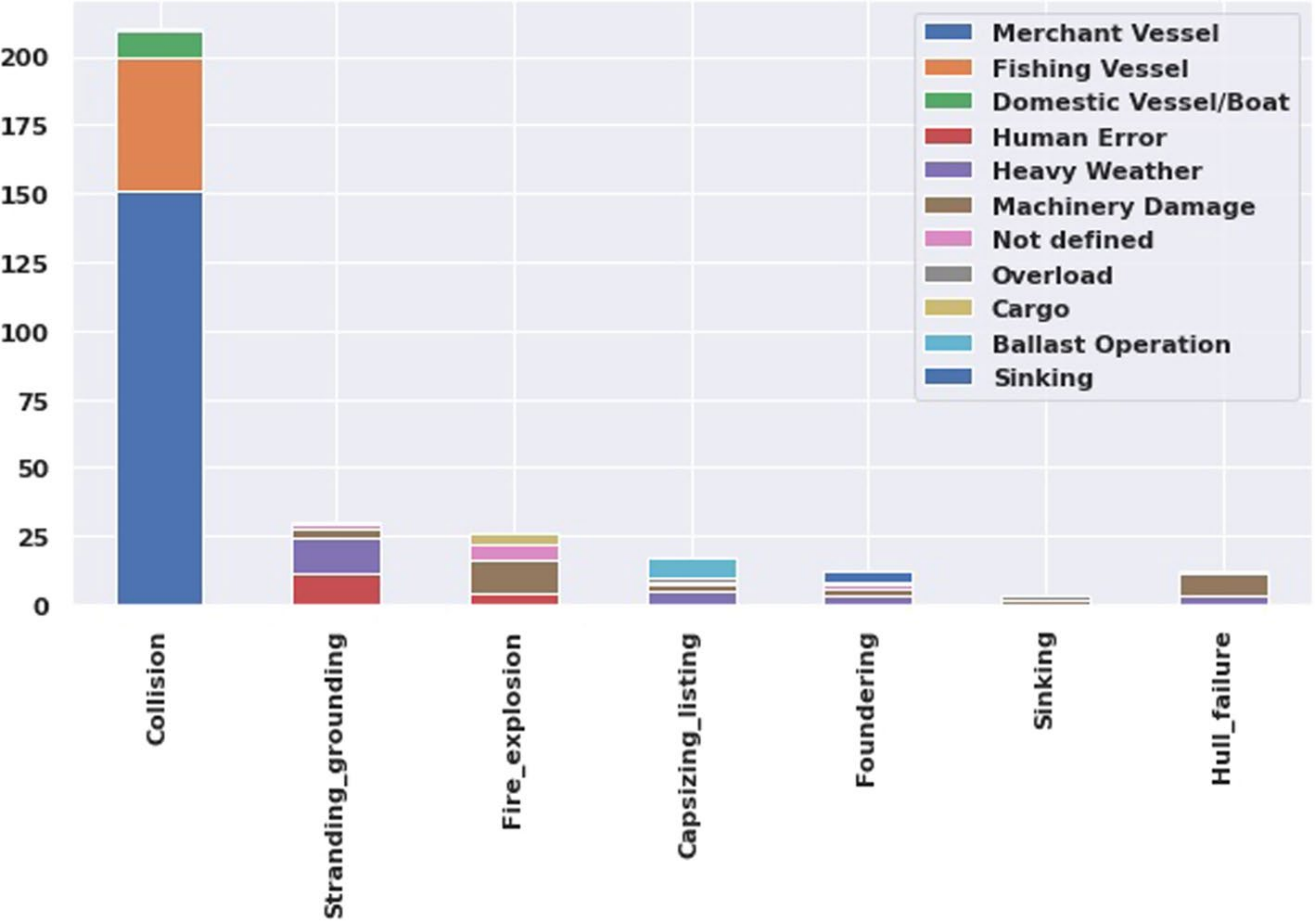
Casualities as per different causes

Figure 2 shows different types of casualties with causes, where the most frequent casualty is Collision between the merchant vessels.

**Fig. 3:**
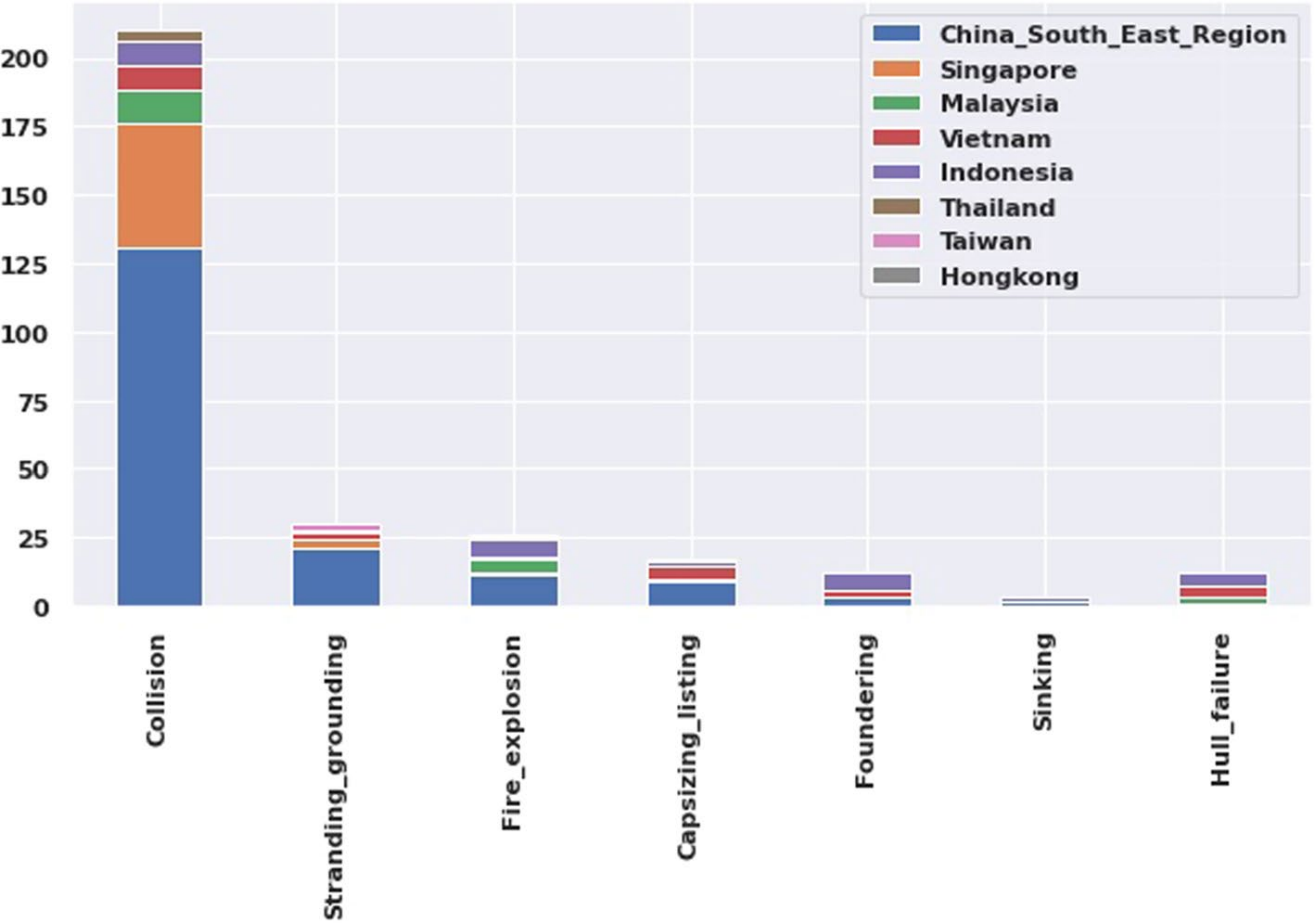
Casualties as per different cost

Figure 3 presents different types of casualties following the coast. The bar chart shows that the South-East region of China is the most collision prone area and the ‘Grounding’ also high in this region. It is interesting that, despite having the lowest casualty rate, the Malaysia coast is accountable for collision and fire after China and Singapore. The collision is the most frequent casualty in China, Singapore, Malaysia, Vietnam, Indonesia, and Thailand.

**Fig. 4:**
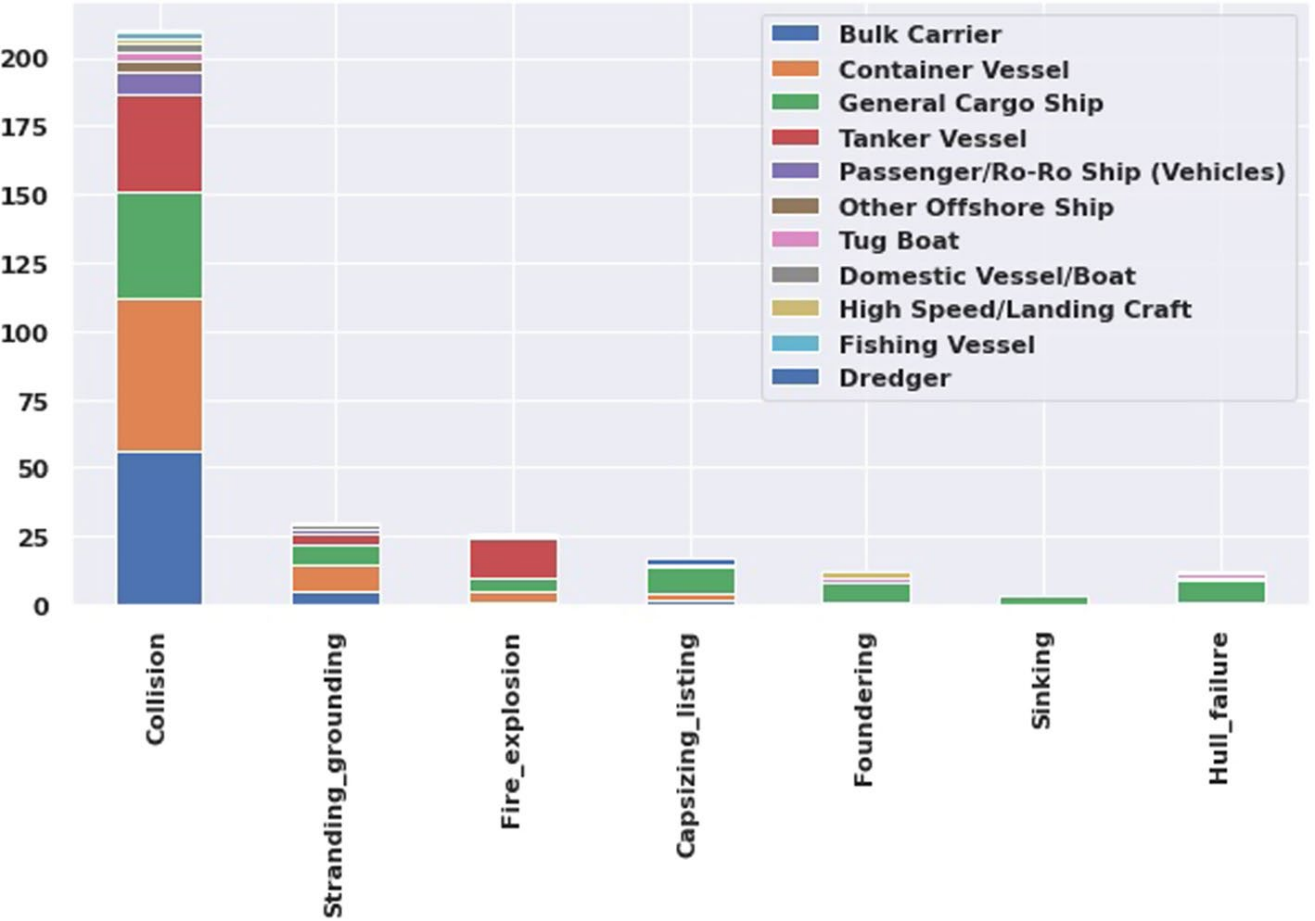
Casualties as per different types of vessels

Figure 4 presents different types of casualties as per vessel type. Maximum collision is occurred in bulk and container vessels where the tanker is accountable for fire or explosion casualty.

**Fig. 5:**
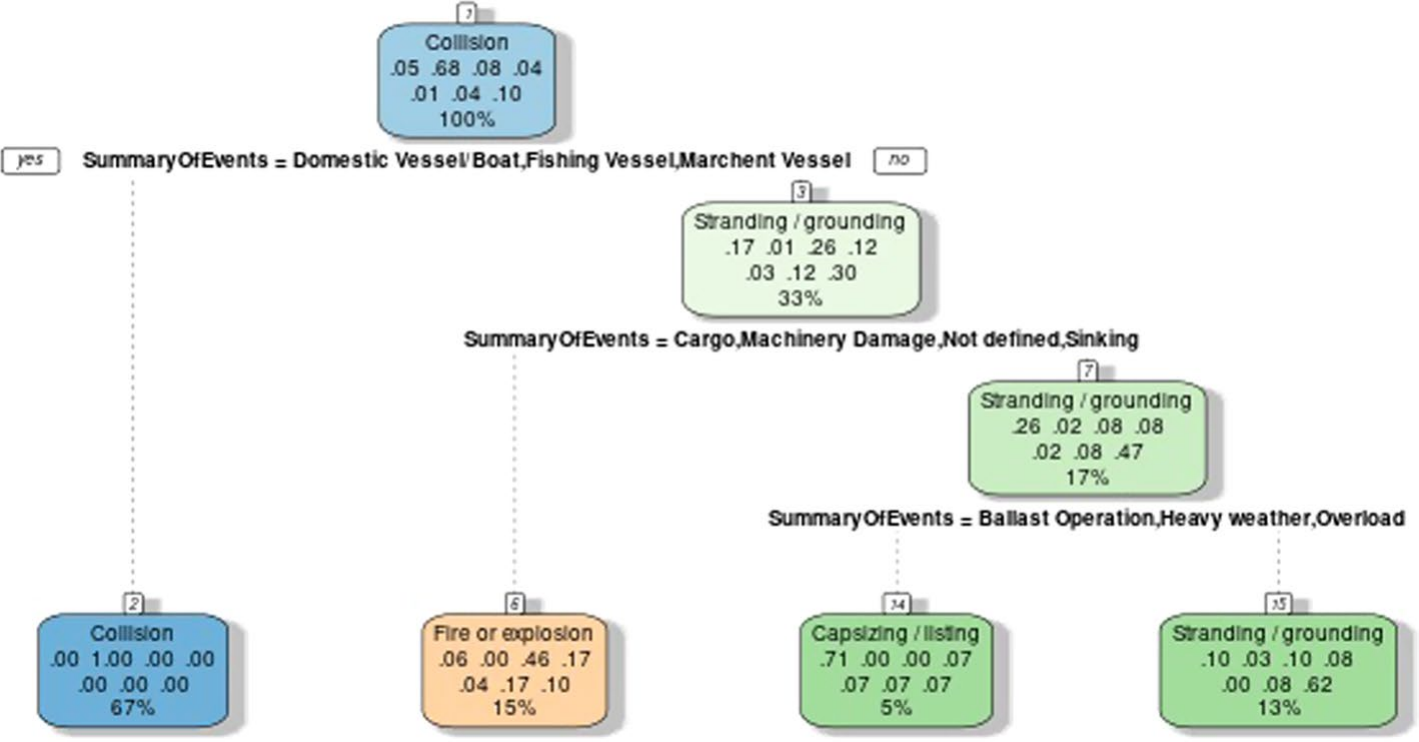
Decision tree of the different types of casualties with cause

Figure 5 presenting decision trees that sort out the most frequent casualty cases. The GISIS has provided information on a variety of casualties. The Decision tree analysis calculated by the R program shows four major types of casualties: Collision, Fire or Explosion, Capsizing/Listing, Stranding/Grounding. In this study, we will follow the decision tree to set our Analytic Hierarchy Process (AHP) criteria.

### MCDA-AHP method for analysis suitable autonomous vessel:

**Fig. 6:**
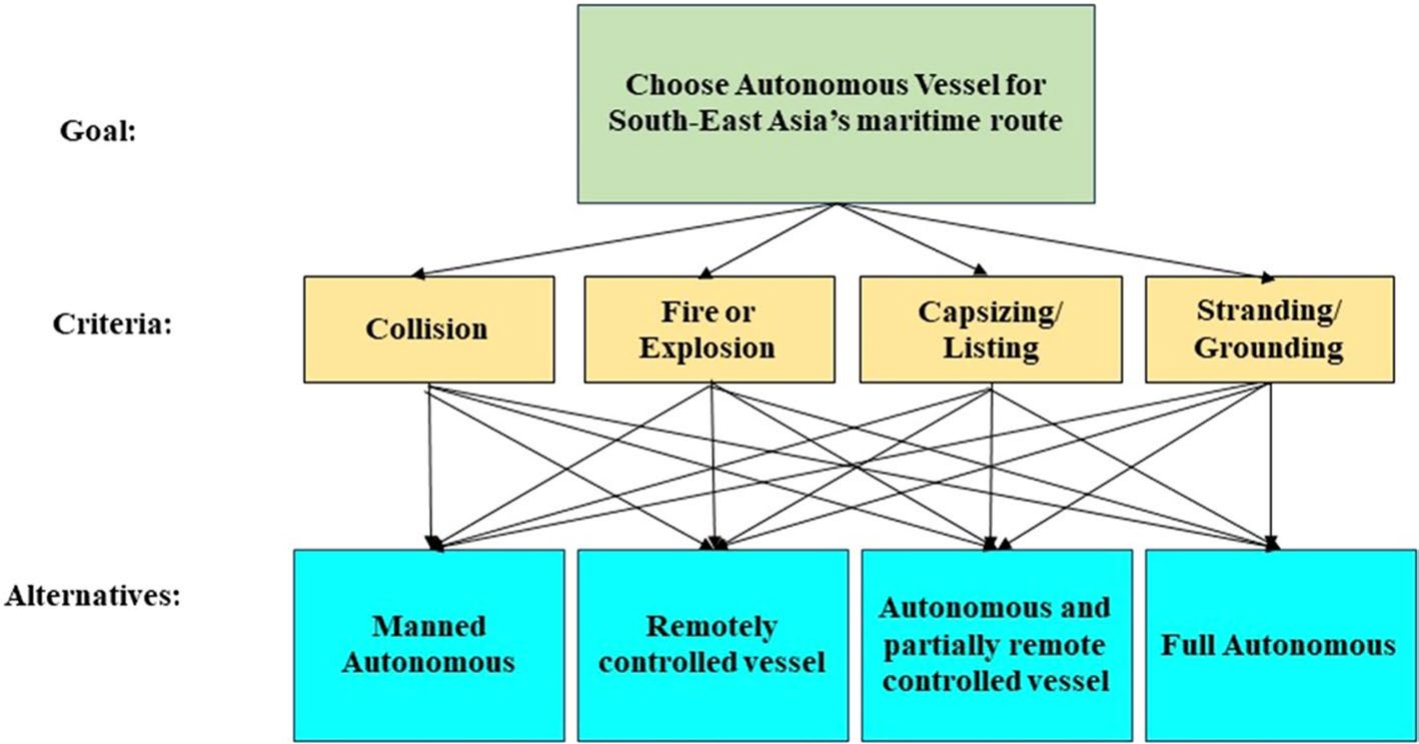
AHP-MCDA application

Analytical Hierarchy Process (AHP) and Multi-Criteria Decision Analysis (MCDA) have been applied in this study. Figure 6, presenting the analysis model of Goals, criteria, and Alternatives. Python 3 was used to complete the model using Topsis from the scikitmcda library. The following steps are followed to conduct the research:

1. Construct a pair-wise matrix of the casualties for AHP:
  The comparison between the criteria was identified using the result of the Fig. 5 decision tree. For the comparison matrix of i and j, the following equation is required to satisfy the equation -2. For this study, ${\omega }_{i}$ or ${\omega }_{j}$ will be the percentage of casualty according to Fig. 5.
  Table 3 represents the pair-wise matrix of the casualties.
2. Calculate Weight matrix:
  Table 4 shows the calculated weight co-efficient for criteria, derived from ‘Pair wise Comparison matrix’.
3. Construct a Numerical decision matrix based on the survey:
  Table 5 shows the ‘Linguistic variables and equivalent numerical values and Table 6 shows the ‘Numerical decision matrix’. As per the survey result, the ‘Numerical decision Matrix’ of the alternatives is constructed. The pattern of Table 5 is used to create the Table 6 matrix.
4. Normalize the decision matrix
  Table 7 shows the ‘Normalized matrix of Alternative’.
5. Calculate weighted normalize decision matrix:
  Table 8 shows the Weighted matrix of the alternatives.
6. Rank the alternatives:

**Table 3:** Pair wise comparison matrix

	C1	C2	C3	C4
C1	1	5	6	9
C2	1/5	1	2	3
C3	1/6	2	1	2
C4	1/9	1/3	1/2	1

**Table 4:** Weight for criteria

Criteria	Weight
Collision	0.659994498441225
Fire or explosion	0.17305153126719236
Capsizing/listing	0.10613729445565132
Stranding/grounding	0.06081667583593128

**Table 5:** Linguistic variables and equivalent numerical values

Linguistic variables	Numerical value
Highly recommended	4
Moderately recommended	3
Recommended	2
Less Recommended	1

**Table 6:** Numerical decision matrix

Alternatives	Collision	Fire or explosion	Stranding/grounding	Capsizing/listing
Manned autonomous	4	4	4	4
Remote control	3	3	3	3
Autonomous and partial remote control	2	2	2	2
Full autonomous	1	1	1	1

**Table 7:** Normalized matrix of Alternatives

Alternatives	Collision	Fire or explosion	Stranding/grounding	Capsizing/listing
Manned autonomous	0.730297	0.730297	0.730297	0.730297
Remote control	0.365148	0.365148	0.365148	0.365148
Autonomous and partial remote control	0.547723	0.547723	0.547723	0.547723
Full autonomous	0.182574	0.182574	0.182574	0.182574

**Table 8:** Weighted matrix of alternatives

Alternatives	Collision	Fire or explosion	Stranding/grounding	Capsizing/listing
Manned autonomous	0.481992	0.126379	0.0775117	0.0444142
Remote control	0.240996	0.0631895	0.0387559	0.0222071
Autonomous and partial remote control	0.361494	0.0947842	0.0581338	0.0333107
Full autonomous	0.120498	0.0315947	0.0193779	0.0111036

This is the final stage where the alternatives are ranked.

Table 9 demonstrates the suitable autonomous vessel for Southeast Asia's route, surveying the opinion of seafarers. The result shows that seafarer thinks that in this dense traffic region where collision is the main casualty, manned autonomous is the appropriate autonomous vessel model to ensure safe navigation.

**Table 9:** Ranking of alternatives

Alternatives	Performance score	Rank
Manned autonomous	1.00	1
Remote control	0.33	3
Autonomous and partial remote control	0.667	2
Full autonomous	0	4

## Discussion

The autonomous vessel's concept catches the world's concern for several reasons, not only for technological advancement, but also because this vessel is considered sustainable shipping. In the shipping industry, autonomous adoption can reduce operational costs and reduce CO_2_ and NO_x_ emissions (Mou et al. 2021). The study conducted a survey on seafarers who has navigation experience. Vessel navigation is a process where some unique and unidentified situations cannot be covered by rules or regulation, own experience and sense achieve the most priority. Nevertheless, several factors can affect safe navigation, such as geographical position, traffic density, weather, currented. Skilled navigators decide by apprising the circumstances and plan following the rules and regulations. Southeast Asia is included in the leading trade corridors of the Maritime Silk Road of China. It is at the pivot point of the world's maritime transportation where the Covid-19 pandemic could not reduce the traffic density (March et al. 2021). Rapid trade growth is apparent between the south-Asian countries, and the most prominent maritime trade flows are among Singapore, Malaysia, Indonesia, and Thailand (Trace et al. 2009). Due to this reason, navigation with caution is essential for this section. In this region, the descriptive statistics show that the most frequent casualties are- collisions, Fire or explosion, Stranding/grounding, Capsizing/listing. Among them, the highest casualty cases are related to collisions. The rules of the road, International Regulations for Preventing Collisions at Sea (COLREG), provide the set of rules for establishing good seamanship to prevent a collision. The implementation of action as per COLREG between manned and unmanned vessels is considered the main challenge for autonomous shipping (Felski and Zwolak 2020).

In order to prevent casualties, accident cause assessment is required for safe navigation planning. The common reasons behind maritime accidents are 60% for human error and 19% for machinery damages (Demirel and Bayer 2015). This study assessed 311 cases from IMO’s GSIS record, and the report reveals that maximum the collision cases are associated between merchant vessels, and in some cases, with the fishing vessels. Heavy weather and human error are the leading cause of the vessel grounding in China and Singapore. In fire explosion events, machinery damage is the main cause, and the ballast operation is responsible for capsizing in many cases.

The autonomous vessel will navigate on behalf of the human brain, due to this reason, experienced seafarers can adequately analyze the required capability of prevention of collision at sea. In the autonomous vessel, a programmed or self-learning algorithm will enable the vessel to take any decision, in which programmed algorithms will follow the COLREG with other regulations, and the self-learning algorithm will be based on the machine learning when COLREG and regulation do not cover the event (Flokkou 2021).The overall result is interesting, as the MCDA analysis result put the 'Manned autonomous' in the first rank and ‘Autonomous and partial remote control' in the second, where ‘Remote control’ and Full Autonomous are in 3rd and last position. It is evident that seafarers rely on human decisions and are interested in continuous operation with Artificial Intelligence. They prefer human–machine collaboration in Southeast Asia's navigation. Needless to say, the weather of south-east Asia is not preferable, unpredicted heavy weather happens there several times annually (Shimada 2019). It is assumed that, navigation in harsh weather and complex geographic area are the reasons for non-reliability on fully autonomous (Ziajka-Poznańska and Montewka 2021). In remote-controlled and partially remote-controlled with AI navigational aid require Shore Control Center (SCC) (Porathe 2014). A technically educated and a trained Shore Control Center (SCCO) operator is required to operate SCC (Saha 2021). To ensure safe navigation, SCCO needs technical knowledge along with management skills. (Saha xxxx). Human-autonomous collaboration can improve autonomous vehicle design and vessel operation by reducing the risk of collision (Thieme and Utne 2017). In order to maintain safe navigation in the South-East Asian route, seafarers are suggesting artificially intelligent equipment with human decision-making.

## Recommendation and conclusion

Implementation and development of autonomous vessels is the growing interest in the shipping industry. Though artificial intelligence is playing an ever-increasing role in the vehicle sector worldwide, for unmanned vessel development, the technology is required to supervise more. Especially during the navigation in such dense traffic and an unpredicted heavy weather region like South-East Asia. As the autonomous vessel will contribute in sustainable shipping, we cannot resist its establishment. The author tried to detect the most suitable autonomous mode for this region from this view. Also suggested the following recommendation in order to implement the maritime unmanned surface ship in South-East Asia:

1. As the maximum passage in this region is a narrow channel, a developed algorithm regarding the narrow channel, TSS (Traffic Separation Scheme), fairway should be taken into consideration.
2. As the current, the set, wind speed and wind direction differ from sea to sea, the vessel's algorithms for steering properties are required to be correlated with several weather circumstances.
3. The fishing vessel is another problem during the navigation in this region. The machine requires algorithm which can detect the fishing net, line, trawlers, a small country boat accurately.
4. As the autonomous technology is innovative and state-of-the-art, sufficient human resources are required to maintain the operation (Kim and Mallam 2020). Therefore, the maritime training institute should start the autonomous vessel familiarization course.
5. European Countries have more collaboration among them in the research and development project of the autonomous vessel than Asian countries. On the other hand, Asian countries adjacent to the Indian Ocean, the Bay of Bengal, and China Sea can collaborate in Autonomous vessel development projects.

The autonomous vessel will change the future maritime arena and maritime logistics sector. Reducing the operational cost and carbon emission, the concept will help the shipping industry to attain the sustainable goal. On the other hand, how the unmanned machine will work, still now it is in doubt. Although the Autonomous projects are still at the oceangoing vessel's development level, some success stories are happening in inland waterways. Southeast Asia is the gateway of Asian trade exchange and at the central point of the global maritime supply chain. Due to this reason, marine traffic is high, and collision is the most frequent accident in this area. Before launching any autonomous vessel in this region, it is required to assess that the vessel is competent to control any casualty like- collision, fire or explosion, grounding, and listing. The study accumulates seafarers' responses to choose the best autonomous vessel alternative. Analyzing with professional knowledge and personal experience, the seafarers suggested the Manned autonomous and the Partially remote-controlled vessel for navigation in Southeast Asia’s maritime route. This study focused on the vessel’s alternative and technical direction is not provided. Further study is suggested from this gap where an algorithm will be developed, especially assessing this region's weather, geographical characteristics, and route nature. Along this, the economic benefits of implementing ‘Autonomous Vessel’ in South-East Asia’s maritime route have not been calculated in this research, further research is required to cover this aspect. This study is beneficial for the decision maker, maritime researcher and shipping companies in investigating the best model of the autonomous vessels for South-East Asia’s maritime route and any dense traffic route, also the study suggested some factors which are important to take in consideration during the making of operation algorithm. The research will contribute in marine engineering and the ship management sector.

## Data Availability

The datasets generated and analyzed during the current study are available in the followings- 1. Maritime Accident Data: Published: 18 January 2022|Version1|https://doi.org/10.17632/z2v2t6dhd6.1. 2. Sample Response: Published: 18 January 2022|Version 1|https://doi.org/10.17632/mfw3t7vyfr.1

## Abbreviations

AAWA: Autonomous waterborne applications initiative
AHP: Analytical hierarchy process
AI: Artificial intelligence
COLREG: International regulations for preventing collisions at sea
GISIS: Global integrated shipping information system
IMO: International maritime organization
IoT: Internet of Things
LIDAR: Light detection and ranging
MASS: Maritime autonomous surface ships
MCDA: Multi criteria decision analysis
MUNIN: Maritime unmanned navigation through intelligence in networks
UNCTAD: United nations conference on trade and development
